# Annotations

**Published:** 1904-04-16

**Authors:** 


### Annotations.

### The London Polyclinic.

The annual meeting of the Medical Graduates' College and Polyclinic, which was held recently, though much less exciting than some recent experiences at the same institution, disclosed a state of affairs eminently satisfactory to those interested in post-graduation teaching in London. It is an open secret that the financial policy of the Polyclinic has during the last few years been the cause of much adverse comment, and the justification of this was provided by the admission of the council in the autumn of last year that they had to contemplate a deficit of some £2,000. Fortunately that crisis was met by resolute action. Under the stimulus of vigorous criticism, the council addressed itself to the task of raising this money and to the revision of the entire financial policy of the institution. These decisions involved some few resignations among the responsible officials, but they were emphatically endorsed by the general sense of the members, and have so far been carried into successful operation. Hence the treasurer is now able to report a much more satisfactory state of the college finances, and we understand that during the present year upwards of a hundred new members have been enrolled. It is evident, too, from the list of lectures and cliniques officially intimated that the council is awake to the advisability of conducting the Polyclinic on a broad and catholic basis, so that the teaching offered there may fully represent the clinical resources of the Metropolis. In this respect the institution has a unique opportunity, and it would have been disappointing to find that the management had failed to recognise this. At the annual meeting the report of the council was unanimously adopted, and after the usual votes of thanks to the retiring members, the following gentlemen were elected to serve on the council, namely, Mr. Lenthal Cheatle, C.B., Mr. Jackson Clarke, Dr. Luff, Dr. Drummond Morier, and Dr. Campbell Thomson.

### The Charity Voting Reform Association.

We have frequently had occasion to draw attention to the evils and hardships which are entailed by the system of discriminating between the rival candidates for entry to charitable institutions by a majority of votes. Happily, this method of selection is rapidly giving place to the more reasonable plan of choosing the worthiest candidates to fill the vacancies which occur. The chief contention of those who uphold the voting system is that it helps to bring in subscriptions. But it has been nothing less than an injustice to the public sense of benevolence to assume that the supporters of a charity will not subscribe unless they receive a *quid pro quo*. The fallacy of this assumption has been amply demonstrated by the fact that those institutions which have wisely abandoned the principle of subscribers' privileges, so far as votes are concerned, for a juster and more humane system, have not suffered in consequence of adopting the latter policy. The present movement to abolish the voting system altogether owes its initiation and success to the energies of the Charity Voting Reform Association. As they say in their report, which we have before us, the voting system "is irrational, since it ensures benefits rather to those who have influence and friends, than to the friendless and forlorn." More than one instance has come to our knowledge where great hardship has been entailed on a candidate who has not been fortunate enough to have the advice and help of influential friends and who in consequence has failed to obtain a sufficient number of votes, until worn out by misery and disappointment, he has died without ever obtaining the advantages of charitable institutions which were especially intended for the benefit of similar cases. Anyone to whom the question appeals may become a member of the Charity Voting Reform Association by the payment of an annual subscription of five shillings, and so take an active share in abolishing what we believe to be a very cruel method of administering charity. The address of the Association is 36 West Street, Charing Cross Road, W.C.

### The Right to Prescribe.

The habit and custom of the British public in accepting medical advice and treatment from non-medical advisers may be difficult to defend on logical or prudential grounds, but the practice exists, and any attempt to prevent it by legal restraints is not within the sphere of practical politics. Naturally members of the medical profession have a keen appreciation of the possible dangers of the course here indicated, and it is within their right or even their duty to remark on these dangers. But such criticism should not suggest that the medical profession enjoys an exclusive or prescriptive right to undertake the treatment of disease. In this country there is certainly no such right. If the non-medical adviser is to be attacked he should be attacked, not on the ground that he is invading some special professional preserve, but because his action suggests his ability to discharge functions for which he is not competent, and because such a proceeding is prejudicial to the public interest. In some recent discussions on the respective provinces of medicine and pharmacy this consideration has at times been lost sight of, with the result that individual advocates have seemed to assert that non-qualified practice is mainly to be condemned as an invasion of the rights of the medical profession. This it may be suggested is a mistake both in substance and in form. Equally mistaken is the notion that public opinion will sanction penal legislation to prevent all but qualified practitioners from prescribing for the relief of the complaints of the public. Pharmacists are commonly reputed to be special sinners in this respect, and without question some of them do assume responsibilities for which they have no warrant. The hope of improvement lies rather in the education of public opinion than in the resources of law. In this education pharmacists who respect their calling may be expected to take an adequate share. But the hope that Parliament will sanction the infliction of a penalty upon any person not being a qualified medical practitioner who suggests a medicinal remedy for what is, or what appears to the sufferer to be, a trifling ailment, is a vain delusion. Freedom, even to act unwisely, is too deeply rooted in the national fibre to permit such an invasion of individual choice.

